# *N*,*N*-Bis(cyano­meth­yl)nitrous amide

**DOI:** 10.1107/S1600536810017265

**Published:** 2010-05-15

**Authors:** Yuan Zhang, Meng Ting Han

**Affiliations:** a Ordered Matter Science Research Center, College of Chemistry and Chemical Engineering, Southeast University, Nanjing 211189, People’s Republic of China

## Abstract

In the title compound, C_4_H_4_N_4_O, both H atoms bonded to one methyl­ene C atom are involved in C—H⋯N hydrogen-bonding inter­actions; one of the inter­actions results in dimers of the title mol­ecule lying about inversion centers in *R*_2_^2^(12) motifs and the other forms chains of mol­ecules lying along the *c* axis.

## Related literature

For background to ferroelectric compounds, see: Haertling (1999 ▶); Homes *et al.* (2001 ▶). For related structures, see: Adolf *et al.* (1996 ▶); Kaida *et al.* (1990 ▶). For graph-set notation, see: Bernstein *et al.* (1994 ▶).
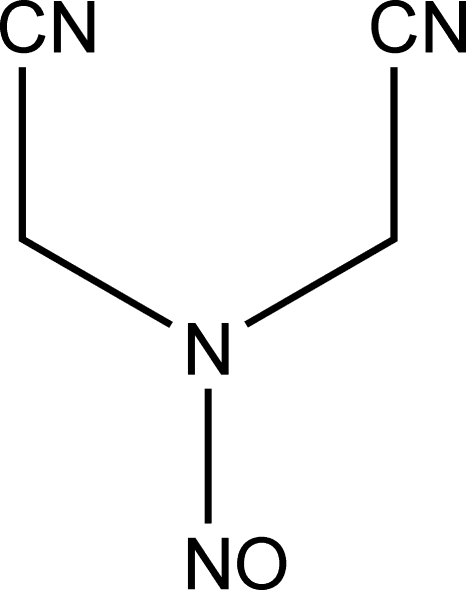


## Experimental

### 

#### Crystal data

C_4_H_4_N_4_O
*M* *_r_* = 124.11
Monoclinic, 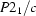

*a* = 6.5622 (13) Å
*b* = 8.9765 (18) Å
*c* = 11.008 (4) Å
β = 108.55 (3)°
*V* = 614.7 (3) Å^3^
*Z* = 4
Mo *K*α radiation
μ = 0.10 mm^−1^
*T* = 293 K
0.20 × 0.20 × 0.20 mm

#### Data collection

Rigaku Mercury2 diffractometer
Absorption correction: multi-scan (*CrystalClear*; Rigaku, 2005 ▶) *T*_min_ = 0.742, *T*_max_ = 1.000
6154 measured reflections
1408 independent reflections
1094 reflections with *I* > 2σ(*I*)
*R*_int_ = 0.059

#### Refinement

*R*[*F* ^2^ > 2σ(*F* ^2^)] = 0.050
*wR*(*F* ^2^) = 0.162
*S* = 1.05
1408 reflections
83 parameters
H-atom parameters constrained
Δρ_max_ = 0.20 e Å^−3^
Δρ_min_ = −0.20 e Å^−3^

### 

Data collection: *CrystalClear* (Rigaku, 2005 ▶); cell refinement: *CrystalClear*; data reduction: *CrystalClear*; program(s) used to solve structure: *SHELXS97* (Sheldrick, 2008 ▶); program(s) used to refine structure: *SHELXL97* (Sheldrick, 2008 ▶); molecular graphics: *SHELXTL* (Sheldrick, 2008 ▶); software used to prepare material for publication: *SHELXTL*.

## Supplementary Material

Crystal structure: contains datablocks I, global. DOI: 10.1107/S1600536810017265/pv2279sup1.cif

Structure factors: contains datablocks I. DOI: 10.1107/S1600536810017265/pv2279Isup2.hkl

Additional supplementary materials:  crystallographic information; 3D view; checkCIF report

## Figures and Tables

**Table 1:** Hydrogen-bond geometry (Å, °)

*D*—H⋯*A*	*D*—H	H⋯*A*	*D*⋯*A*	*D*—H⋯*A*
C3—H3*B*⋯N2^i^	0.97	2.50	3.450 (2)	165
C3—H3*C*⋯N1^ii^	0.97	2.62	3.183 (2)	117

Symmetry codes: (i) 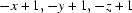; (ii) 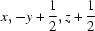.

**Fig. 1.**
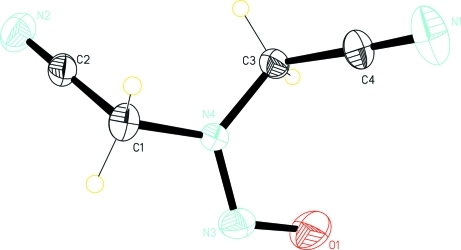
Perspective drawing of the title compound with displacement ellipsoids drawn at the 30% probability level.

**Fig. 2.**
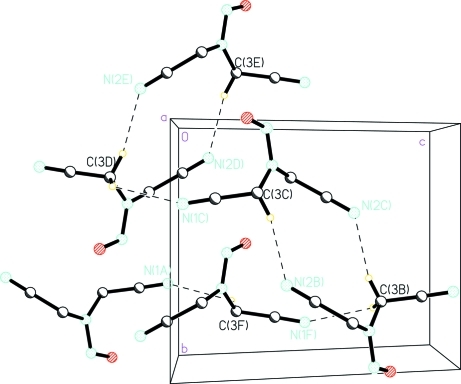
The crystal packing of the title compound viewed along the a-axis showing H-bondings interactions; the H atoms not involved in H-bonds have been ommitted for clarity.

## supplementary crystallographic information

### Comment

At present, much attention in the field of ferroelectric materials is focused on developing ferroelectric organic or inorganic compounds (Haertling *et al.*, 1999; Homes *et al.*, 2001). It has been reported that *N*,*N*-bis(cyanomethyl)nitramide crystallizes in space group (C 2) at room temperature (Adolf *et al.*, 1996), a noncentrosymmetric space group is required for ferroelectric behavior. Its ferroelectric property still needs to be further confirmed by many experiments, such as dielectric measurements and DSC to varify the permittivity anomaly, phase transition, etc. For this reason, we have synthesized the title compound to investigate its physical properties. The dielectric constant of the title compound as a function of temperature indicates that the permittivity is basically temperature-independent (dielectric constant = 3.2 to 5.6), suggesting that this compound should not be a real ferroelectric or there may be no distinct phase transition within the measured temperature range. Similarly, below the melting point (308 K) of the compound, the dielectric constant as a function of temperature also goes smoothly, and there is no dielectric anomaly observed. Herein, we report the synthesis and crystal structure of the title compound.

The bond distances and bond angles in the title compound agree very well with the corresponding distances and angles reported for a closely related compound (Kaida *et al.*, 1990); both cyanic groups are linear (Fig. 1). It is interesting to note that both H-atoms bonded to only one methylene carbon (C3) are involved in hydrogen bonding interactions of the type C—H···N, C3—H3B···N2 hydrogen bonds result in dimers of the title molecule lying about inversion centers in R_2_^2^(12) motifs in graph set notation (Bernstein *et al.*, 1994) while C3—H3C···N1 interactions result in chains of molecules lying along the *c*-axis (Tab. 1, Fig. 2). Dipole–dipole and van der Waals interactions are effective in the molecular packing.

### Experimental

A solution of sodium nitrite (2.3 g, 33 mmoles) in water (10 ml) was added at 291–293 K to a solution of 2,2'-azanediyldiacetonitrile hydrochloride (1.7 g, 28 mmoles) in water (30 ml). The mixture was heated for 1.5 h at 313–323 K and allowed to stand for 12 h at 293 K. The title compound as nitroso derivative, was extracted with ether, the ether solution was evaporated. Single crystals suitable for X-ray diffraction analysis were obtained from slow evaporation of an ethyl acetate solution of the title compound.

### Refinement

H atoms were positioned geometrically and refined using a riding model, with C—H = 0.97 Å and *U*_iso_(H) = 1.2U_eq_(C).

### Crystal data

**Table:**

C_4_H_4_N_4_O	*F*(000) = 256
*M**_r_* = 124.11	*D*_x_ = 1.341 Mg m^−^^3^
Monoclinic, *P*2_1_/*c*	Mo *K*α radiation, λ = 0.71073 Å
Hall symbol: -P 2ybc	Cell parameters from 1409 reflections
*a* = 6.5622 (13) Å	θ = 2.3–27.5°
*b* = 8.9765 (18) Å	µ = 0.10 mm^−^^1^
*c* = 11.008 (4) Å	*T* = 293 K
β = 108.55 (3)°	Prism, colorless
*V* = 614.7 (3) Å^3^	0.20 × 0.20 × 0.20 mm
*Z* = 4	

### Data collection

**Table:**

Rigaku Mercury2 diffractometer	1408 independent reflections
Radiation source: fine-focus sealed tube	1094 reflections with *I* > 2σ(*I*)
graphite	*R*_int_ = 0.059
Detector resolution: 13.6612 pixels mm^-1^	θ_max_ = 27.5°, θ_min_ = 3.0°
CCD_Profile_fitting scans	*h* = −8→8
Absorption correction: multi-scan (*CrystalClear*; Rigaku, 2005)	*k* = −11→11
*T*_min_ = 0.742, *T*_max_ = 1.000	*l* = −14→14
6154 measured reflections	

### Refinement

**Table:**

Refinement on *F*^2^	Secondary atom site location: difference Fourier map
Least-squares matrix: full	Hydrogen site location: inferred from neighbouring sites
*R*[*F*^2^ > 2σ(*F*^2^)] = 0.050	H-atom parameters constrained
*wR*(*F*^2^) = 0.162	*w* = 1/[σ^2^(*F*_o_^2^) + (0.1*P*)^2^] where *P* = (*F*_o_^2^ + 2*F*_c_^2^)/3
*S* = 1.05	(Δ/σ)_max_ < 0.001
1408 reflections	Δρ_max_ = 0.20 e Å^−^^3^
83 parameters	Δρ_min_ = −0.20 e Å^−^^3^
0 restraints	Extinction correction: *SHELXL97* (Sheldrick, 2008), Fc^*^=kFc[1+0.001xFc^2^λ^3^/sin(2θ)]^-1/4^
Primary atom site location: structure-invariant direct methods	Extinction coefficient: 0.15 (2)

### Special details

**Table:**

Geometry. All esds (except the esd in the dihedral angle between two l.s. planes) are estimated using the full covariance matrix. The cell esds are taken into account individually in the estimation of esds in distances, angles and torsion angles; correlations between esds in cell parameters are only used when they are defined by crystal symmetry. An approximate (isotropic) treatment of cell esds is used for estimating esds involving l.s. planes.
Refinement. Refinement of *F*^2^ against ALL reflections. The weighted *R*-factor wR and goodness of fit *S* are based on *F*^2^, conventional *R*-factors *R* are based on *F*, with *F* set to zero for negative *F*^2^. The threshold expression of *F*^2^ > σ(*F*^2^) is used only for calculating *R*-factors(gt) etc. and is not relevant to the choice of reflections for refinement. *R*-factors based on *F*^2^ are statistically about twice as large as those based on *F*, and *R*- factors based on ALL data will be even larger.

### Fractional atomic coordinates and isotropic or equivalent isotropic displacement parameters (Å^2^)

**Table:**

	*x*	*y*	*z*	*U*_iso_*/*U*_eq_	
O1	0.1721 (2)	−0.00421 (14)	0.24979 (14)	0.0738 (5)	
N1	0.1213 (3)	0.3227 (2)	0.03826 (14)	0.0730 (6)	
N2	0.6354 (3)	0.36079 (17)	0.61699 (14)	0.0593 (5)	
N3	0.3553 (2)	0.02971 (15)	0.31634 (14)	0.0537 (5)	
N4	0.38510 (18)	0.17474 (12)	0.33544 (10)	0.0345 (4)	
C2	0.6201 (2)	0.29919 (17)	0.52436 (14)	0.0411 (4)	
C1	0.6039 (2)	0.22069 (18)	0.40327 (13)	0.0419 (4)	
H1A	0.6543	0.2860	0.3487	0.050*	
H1B	0.6962	0.1336	0.4222	0.050*	
C4	0.1609 (2)	0.30241 (19)	0.14509 (15)	0.0439 (4)	
C3	0.2111 (2)	0.28025 (16)	0.28328 (13)	0.0405 (4)	
H3B	0.2504	0.3752	0.3264	0.049*	
H3C	0.0835	0.2446	0.3006	0.049*	

### Atomic displacement parameters (Å^2^)

**Table:**

	*U*^11^	*U*^22^	*U*^33^	*U*^12^	*U*^13^	*U*^23^
O1	0.0564 (9)	0.0523 (9)	0.0991 (11)	−0.0192 (6)	0.0056 (7)	−0.0124 (6)
N1	0.0569 (11)	0.1157 (16)	0.0448 (9)	0.0196 (9)	0.0140 (7)	0.0171 (8)
N2	0.0655 (11)	0.0593 (10)	0.0484 (8)	−0.0107 (8)	0.0113 (7)	−0.0105 (7)
N3	0.0509 (9)	0.0336 (8)	0.0697 (10)	−0.0029 (6)	0.0094 (7)	−0.0008 (6)
N4	0.0342 (7)	0.0298 (7)	0.0356 (7)	0.0010 (5)	0.0056 (5)	0.0001 (4)
C2	0.0369 (8)	0.0392 (8)	0.0407 (8)	−0.0047 (6)	0.0032 (6)	0.0029 (6)
C1	0.0357 (9)	0.0485 (9)	0.0387 (8)	−0.0044 (7)	0.0076 (6)	−0.0030 (6)
C4	0.0339 (8)	0.0545 (9)	0.0390 (9)	0.0035 (7)	0.0058 (6)	0.0048 (6)
C3	0.0449 (9)	0.0372 (8)	0.0352 (8)	0.0099 (6)	0.0067 (6)	−0.0017 (6)

### Geometric parameters (Å, °)

**Table:**

O1—N3	1.2305 (18)	C2—C1	1.481 (2)
N1—C4	1.135 (2)	C1—H1A	0.9700
N2—C2	1.136 (2)	C1—H1B	0.9700
N3—N4	1.3233 (18)	C4—C3	1.464 (2)
N4—C1	1.4516 (18)	C3—H3B	0.9700
N4—C3	1.4539 (17)	C3—H3C	0.9700
			
O1—N3—N4	113.89 (13)	C2—C1—H1B	109.2
N3—N4—C1	115.67 (12)	H1A—C1—H1B	107.9
N3—N4—C3	121.36 (12)	N1—C4—C3	178.53 (19)
C1—N4—C3	122.83 (12)	N4—C3—C4	112.80 (12)
N2—C2—C1	178.83 (17)	N4—C3—H3B	109.0
N4—C1—C2	111.97 (13)	C4—C3—H3B	109.0
N4—C1—H1A	109.2	N4—C3—H3C	109.0
C2—C1—H1A	109.2	C4—C3—H3C	109.0
N4—C1—H1B	109.2	H3B—C3—H3C	107.8

### Hydrogen-bond geometry (Å, °)

**Table:**

*D*—H···*A*	*D*—H	H···*A*	*D*···*A*	*D*—H···*A*
C3—H3B···N2^i^	0.97	2.50	3.450 (2)	165
C3—H3C···N1^ii^	0.97	2.62	3.183 (2)	117

Symmetry codes: (i) −*x*+1, −*y*+1, −*z*+1; (ii) *x*, −*y*+1/2, *z*+1/2.
